# BEHAVIORAL ACTIVATION FOR FAMILY CAREGIVERS OF PEOPLE WITH DEMENTIA: A SYSTEMATIC REVIEW AND META-ANALYSIS

**DOI:** 10.1093/geroni/igz038.407

**Published:** 2019-11-08

**Authors:** Xinyi Xu, Rick Kwan, Angela Y M Leung

**Affiliations:** 1 The Hong Kong Polytechnic University, Hong Kong, Hong Kong; 2 Centre for Gerontological Nursing, School of Nursing, The Hong Kong Polytechnic University, Hong Kong, Hong Kong

## Abstract

Behavioural activation (BA) aims to increase positive response-contingent environmental reinforcement and help caregivers to engage in pleasant and constructive activities, and therefore improve psychological and physical health among family caregivers of people with dementia (PWD). However, knowledge of the effectiveness of BA in this population remains limited. The current study applied a systematic review and meta-analysis in order to determine the effectiveness of BA among family caregivers of PWD. Literature was searched in PubMed, Medline, CINAHL, Cochrane, Embase and PsycINFO published from March 1988 to March 2018. Seven Randomized Control Trials (RCT)s evaluating the effects of BA in family caregivers of PWD were eligible to be included in this review. Cochrane’s guideline was used in order to measure risk of bias and extract data. A random effects model was used to pool the effect size. Family caregivers of PWD receiving BA that only for caregivers demonstrated a statistically significant reduction in depression (n=3; 311 participants; Cohen’d=0.55; 95% CI: 0.30 to 0.81; P<0.001). BA also had a positive impact on interlukin-6, negative affect of caregiving, relationship satisfaction, dysfunctional thoughts and distress related to neuropsychiatric symptoms of PWD for family caregivers. The available evidence suggests that future studies are needed to focus on better ways of administering BA to family caregivers of PWD, to improve their physical and psychological health. Meanwhile, more RCTs to investigate the effects of BA on psychological and physical health for family caregivers of PWD is needed.

